# Foxp3 overexpression in tumor cells predicts poor survival in oral squamous cell carcinoma

**DOI:** 10.1186/s12885-016-2419-6

**Published:** 2016-07-26

**Authors:** Jing-Jing Song, Si-Jia Zhao, Juan Fang, Da Ma, Xiang-Qi Liu, Xiao-Bing Chen, Yun Wang, Bin Cheng, Zhi Wang

**Affiliations:** 1 Guanghua School of Stomatology, Guangdong Provincial Key Laboratory of Oral Diseases, Sun Yat-sen University, No. 56, Lingyuan West Road, Guangzhou, 510055 China; 2 Chinese Academy of Medical Sciences & Peking Union Medical College, Beijing, China

**Keywords:** Oral squamous cell carcinoma (OSCC), Foxp3, Five years overall survival, Relapse-Free Survival(RFS)

## Abstract

**Background:**

Forkhead Box P3 (Foxp3) is a regulatory T cells marker, and its expression correlates with prognosis in a number of malignancies. The aim of this study is to determine the relationship of Foxp3 expression with clinicopathological parameters and prognosis in oral squamous cell carcinoma (OSCC).

**Methods:**

Foxp3 expression was examined using immunohistochemistry (IHC) in paraffin-embedded tissue samples from 273 OSCC patients. Statistical analysis was performed to evaluate the associations between Foxp3 expression, the clinicopathologic characteristics and prognostic factors in OSCC.

**Results:**

Foxp3 protein expression was significantly associated with lymph node metastasis (*P* <0.01). Both univariate and multivariate analyses revealed that Foxp3 was an independent factor for both 5 years overall survival (OS) and relapse-free survival (RFS) (both *P* <0.01). Patients with Foxp3 overexpression had shorter OS and RFS.

**Conclusions:**

Our results determined that elevated Foxp3 protein expression was a predictive factor of outcome in OSCC and could act as a promising therapeutic target.

## Background

Consisting 90 % of oral malignancies, oral squamous cell carcinoma (OSCC) is one of the leading causes of cancer-related death worldwide due to poor prognosis. The annual estimated occurrence of lip and oral cavity malignancies is around 400,000 cases [1]. Despite a rapidly growing number of treatment options, the 5-year survival rate of OSCC still remains under 60 %. Apart from classification of differentiation and TNM stage there are as yet hardly any predictive factors for prognosis in patients with OSCC. In this situation it is of utmost importance to identify potential molecular markers and targets which provide the necessary condition of accurate prediction of 5 years overall survival (OS) and relapse-free survival (RFS) [2].

Forkhead Box P3 (Foxp3) has played a key role in the immunosuppressive functions in Tregs and a marked molecule of Tregs as well [3]. The presence of Foxp3 expression in cancer cells has been proved to be associated with tumor biological behaviors regulation [4]. A series of studies have illustrated that the mutation or loss of Foxp3 might lead to a high rate of tumor formation, such as developing in mammary [5] or prostatic [6] epithelial tissues, which indicated Foxp3 as an oncosuppressive factor. Moreover, high tumor Foxp3 levels in tumor cells are also found to be related to better outcome of cancer patients of gastric cancer [7] and Her2-positive breast carcinoma [8]. In contrast, only two retrospective studies in patients with tongue squamous cell carcinoma (TSCC) [9] and orohypopharynx squamous cell carcinoma (OHSCC) [10] have demonstrated accordant results that high Foxp3 expression was associated with poor overall survival. Until now, no further studies have been conducted to examine the prognostic significance of Foxp3 expression in OSCC. In the present study, we investigated the tumor cell Foxp3 expression in a large 273 case OSCC cohort and determined the correlation of Foxp3 with clinicopathologic factors, relapse, and prognostic significance of OSCC.

## Methods

### Study population

Patients included in the study were 188 males and 85 females with OSCC treated at the Department of Oral-Maxillofacial Surgery, Stomatology Hospital Affiliated to Sun Yat-sen University between 2002 and 2009. Determination of TNM classification is on basis of the Union for International Cancer Control 2002 standard (UICC2002). The end point of this study is that of 5 year overall survival (OS) and relapse-free survival (RFS). Pathological examination was performed by two independent pathologist according to the 2005 revised World Health Organization classification of tumors.

### Tissue microarrays and IHC testing of patient samples

Tissue microarrays (TMAs) were constructed for 273 patients cohorts using three 2 mm tissue cores collected from areas of OSCC epithelium on each tissue block. Sections were cut into 4 μm thickness, deparaffinized, rehydrated and treated with a peroxidase block. Foxp3 staining procedures was based on manufacturers protocol. Heat-based antigen retrieval was carried out by microwave treatment in 10 mM citrate buffer (pH 6.0). The sections were incubated with mouse monoclonal anti-human Foxp3 (clone AO1042a) diluted 1:200 (Abgent, USA) at 4 °C overnight, followed by incubation with ready to use EnVision HRP-IgG secondary antibody (DAKO, Denmark) for 30 min. Staining was developed using 3, 3′-diaminobenzidine (DAB) as a chromogen substrate. The nuclei were counterstained with Mayer’s hematoxylin.

Predominantly cytoplasmic staining was observed in tumor cells. The immunoreactive score (IRS) was calculated as intensity of the staining reaction multiplied by the percentage of positive cells. Based on the IRS values, Foxp3 were scored as low, medium and high [10]. Representative micrographs indicating that medium and high levels of Foxp3 were found only in malignant tissues, while healthy epithelial tissues displayed only low levels of Foxp3. Consequently according to the HLA & Cancer Component of the 12th IHW [11], the results were confirmed by two experienced pathologists who were blinded to the clinicopathologic data of the patients. The whole sections were repeatly operated in the condition that tissue microarray staining was not typical represented. Negative controls which were treated without primary antibodies were implemented during each experimentation.

### Statistical analysis

The OS rate was the primary endpoint of this study, and the secondary endpoint was the relapse-free survival (RFS) of the OSCC patients. OS was defined as the duration from the date of each patient’s hospitalization to the date of death from any cause or to the censoring of the patient at the date of the last follow-up. RFS was defined as the time from hospitalization to local, regional, distant failure, or other second primary cancer. All statistical analyses were conducted using SPSS 22.0 statistical software. The relationships between Foxp3 expression and the clinicopathological characteristics were using Linear-by-Linear Association test (Age, Differentiation, T stage, N stage, and Clinical stage), and Pearson’s chi-square test (Gender, Smoking, Drinking, and Tumor site). Patient survival was evaluated using the Kaplan-Meier method and compared using Log-Rank test. Univariate and multivariate Cox regression analyses were performed to analyze the survival data. Multiple comparison (Wilcoxon Rank Sum test) was performed to analyze the mean OSCC survival time of Foxp3 staining value. *P*-value <0.05 was considered statistically significant.

## Results

### Association of Foxp3 with lymph node metastasis and other clinicopathological variables

**Fig. 1:**
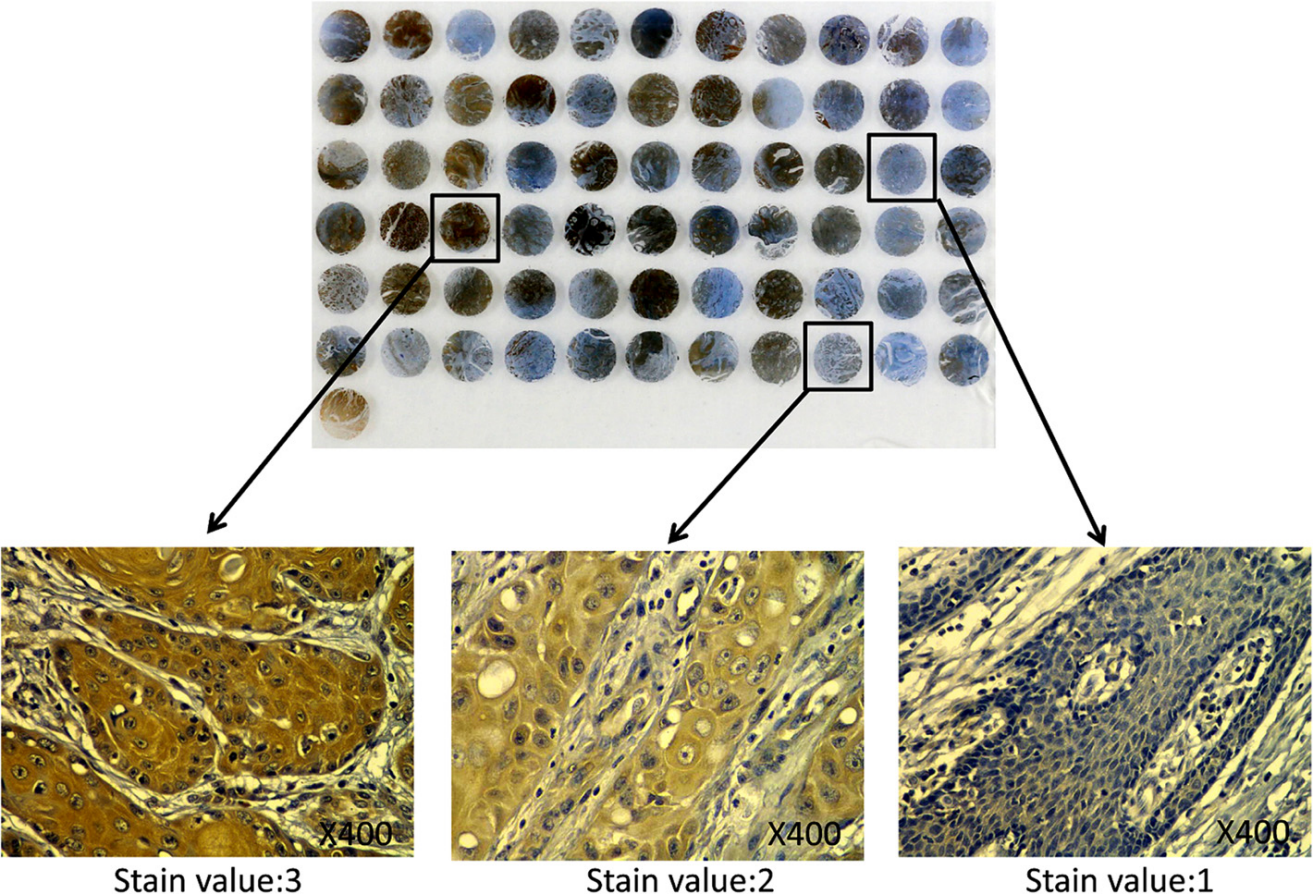
Expression and scoring of Foxp3 in oral squamous cell carcinoma (OSCC) tissue. Representative micrographs from tissue microarray (TMA) cores indicating the low, medium and high cytoplasmic expression in tumor nest

In a cohort of 273 patients with OSCC, Foxp3 expression was determined to be low in 99 (36.3 %), medium in 106 (38.8 %) and high in 68 (24.9 %) patients (Fig. 1). The median age of patients at diagnosis was 60 (range 24–85) and was followed up for an average of 3.58 years. Our analyses showed that the level of Foxp3 in OSCC was significantly correlated with lymph node metastasis (*P* <0.001), but was not associated with age, gender, smoking, drinking, grade of differentiation, tumor site, T stage, Clinical stage (*P* >0.05) (Table 1). Notably, the correlation of Foxp3 with prominent lymph node metastasis positivity suggested a potential role of Foxp3 in increased invasion and metastasis of OSCC.

**Table 1:** Correlation between the Foxp3 expression and clinicopathological characteristics of OSCC

Clinical pathological feature	*N*	Expression of Foxp3			
		Stain value = 1	Stain value = 2	Stain value = 3	*P* Value
		*N* = 99	*N* = 106	*N* = 68	
Age					0.65
<60	133	51 (38.3 %)	49 (36.9 %)	33 (24.8 %)	
≥60	140	48 (34.3 %)	57 (40.7 %)	35 (25.0 %)	
Sex					0.29
Male	188	63 (33.5 %)	74 (39.4 %)	51 (27.1 %)	
Female	85	36 (42.4 %)	32 (37.6 %)	17 (20.0 %)	
Smoking					0.84
Yes	132	47 (35.6 %)	50 (37.9 %)	35 (26.5 %)	
No	141	52 (36.9 %)	56 (39.7 %)	33 (23.4 %)	
Drinking					0.47
Yes	131	45 (34.4 %)	49 (37.4 %)	37 (28.2 %)	
No	142	54 (38.0 %)	57 (40.1 %)	31 (21.9 %)	
Differentiation					0.30
High	200	73 (36.5 %)	82 (41.0 %)	45 (22.5 %)	
Medium	64	23 (35.9 %)	22 (34.4 %)	19 (29.7 %)	
Low	9	3 (33.3 %)	2 (22.2 %)	4 (44.5 %)	
Tumor site					0.23
Gingiva	52	16 (30.8 %)	19 (36.5 %)	17 (32.7 %)	
Tongue	131	50 (38.1 %)	47 (35.9 %)	34 (26.0 %)	
Buccal	61	26 (42.6 %)	24 (39.4 %)	11 (18.0 %)	
Others^a^	29	7 (24.1 %)	16 (55.2 %)	6 (20.7 %)	
T stage					0.63
T1-T2	186	68 (36.5 %)	74 (39.8 %)	44 (23.7 %)	
T3-T4	87	31 (35.6 %)	32 (36.8 %)	24 (27.6 %)	
N stage					0.000***
N0	183	73 (39.9 %)	82 (44.8 %)	28 (15.3 %)	
N1-N3	90	26 (28.9 %)	24 (26.7 %)	40 (44.4 %)	
Clinical Stage					0.09
I-II	142	55 (38.8 %)	59 (41.5 %)	28 (19.7 %)	
III-IV	131	44 (33.6 %)	47 (35.9 %)	40 (30.5 %)	

Stain value = 1, low level of Foxp3 expression; Stain value = 2, medium level of Foxp3 expression; Stain value = 3, high levels of Foxp3 expression

^a^Others included include hard palate, oropharynx and lips

*P* value was determined using the Linear-by-Linear Association test. *,*P*<0.05;**, *P*<0.01;***, *P*<0.001

### Relationship between Foxp3 expression and prognosis in OSCC patients

**Fig. 2:**
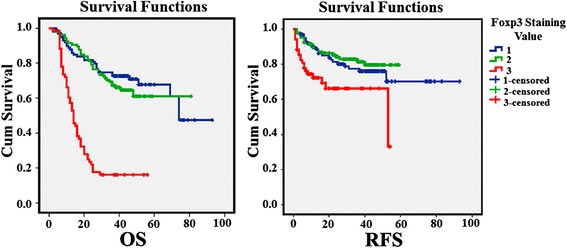
Survival curves of Foxp3 of OSCC patients. It showed that Kaplan–Meier curves of 5 years Overall Survival (OS) and Relapse-Free Survival (RFS) of different Foxp3 staining values. *P*Values among different groups were calculated by the Log-Rank test. There was significant difference between high Foxp3 staining and either of the other two staining grades

To determine whether Foxp3 expression might be a prognostic predictor in OSCC, we examined Foxp3 expression levels and the clinical follow-up information in all 273 patients of OSCC by Kaplan-Meier analysis and Log-Rank test. 126 patients died during the follow-up period, whereas 147 patients were still alive at the end of follow-up. The crude and adjusted relative risks of all-cause mortality in these 273 patients were assessed by univariate and multivariate analyses. As univariate analyses shown in Table 1, the clinicopathological parameters such as N stage, Clinical stage and Foxp3 expression were related to 5-year OS rate, whereas T stage, Clinical stage and Foxp3 expression were related to 5-year RFS rate. Multivariate analyses were used to reveal that T stage, N stage, Clinical stage and Foxp3 expression were the independent risk factors for OS, and Foxp3 expression were the most important risk factor for relapse (Tables 2 and 3; Fig. 2).

**Table 2:** Univariate Analyses of Selected Characteristics with 5 years Overall Survival (OS) and Relapse-free Survival (RFS) among Patients with Oral Squamous Cell Carcinoma (*N* = 273)

Characteristics	5-years OS rate (95 % *CI*)	*P* Value	5-year RFS rate (95 % *CI*)	*P* Value
T stage				
T1-T2	0.415 (0.256–0.574)	0.212	0.652 (0.540–0.764)	0.034*
T3-T4	0.252 (−0.101–0.605)		0.713 (0.448–0.978)	
N stage				
N0	0.470 (0.290–0.650)	0.000***	0.671 (0.548–0.794)	0.584
N1-N3	0.252 (−0.101–0.605)		0.733 (0.623–0.843)	
Clinical stage				
I-II	0.437 (0.249–0.625)	0.012*	0.627 (0.502–0.752)	0.043*
III-IV	0.303 (0.052–0.554)		0.727 (0.527–0.927)	
Foxp3 staining value				
1	0.474 (0.268–0.680)	0.000***	0.701 (0.562–0.840)	0.003**
2	0.611 (0.499–0.723)		0.795 (0.711–0.879)	
3	0.162 (0.074–0.250)		0.330 (0.133–0.793)	

*Abbreviations*: *95 % CI* 95 % confidence interval

*P* value was determined using the Log-rank test. *,*P*<0.05;**, *P*<0.01;***, *P*<0.001

**Table 3:** Multivariate COX Regression analysis on factors for OSCC survival

Characteristics	5-years OS rate		5-year RFS rate	
	HR (95 % *CI*)	*P* Value	HR (95 % *CI*)	*P* Value
T Stage				
T1 or T2	0.547 (0.331–0.904)	0.019*	1.188 (0.483–2.923)	0.707
T3 or T4	Reference		Reference	
N Stage				
N0	0.238 (0.143–0.398)	0.000***	0.614 (0.280–1.347)	0.223
N1-N3	Reference		Reference	
Clinical Stage				
I-II	2.503 (1.348–4.649)	0.004**	2.249 (0.865–5.852)	0.097
III-IV	Reference		Reference	
Foxp3 Staining Value				
1	0.197 (0.123–0.315)	0.000***	0.451 (0.239–0.849)	0.014*
2	0.271 (0.174–0.422)	0.000***	0.371 (0.192–0.714)	0.003**
3	Reference		Reference	

*Abbreviations*: *OS* overall survival, *RFS*, relapse free survival, *HR* hazard ratio, *95 % CI* 95 % confidence interval

*P* value was determined using Cox proportional-hazards model. *,*P*<0.05;**, *P*<0.01;***, *P*<0.001

The results showed that the median overall survival time in patients with high levels of Foxp3 expression (stain value = 3) (14, 2–56 months, *n* = 68) was significantly shorter than that in patients with a low level of Foxp3 expression (stain value = 1) (43, 4–93 months, *n* = 99) (*P* = 0.000) and medium level of Foxp3 expression (stain value = 2) (38, 2–81 months, *n* = 106) (*P* = 0.000). Furthermore, the median relapse-free survival time was markedly longer in the low Foxp3 expression group (39, 2–93 months, *n* = 99) (*P* = 0.000) and medium Foxp3 expression group (36, 2–59 months, *n* = 106) (*P* = 0.000) compared to that in the high Foxp3 expression group (11, 1–54 months, *n* = 68). These results indicated that patients with high levels of Foxp3 expression have a worse prognosis than those with low levels of Foxp3 expression (Table 4). The hazard ratio of 5 years OS for low and medium Foxp3 expression group are 0.197 (*P* = 0.000) and 0.271 (*P* = 0.000), respectively, while that of relapse-free survival (RFS) for low and medium Foxp3 expression group are 0.451 (*P* = 0.014) and 0.371 (*P* = 0.003) taking high Foxp3 expression group as reference (Table 3).

**Table 4:** Multiple comparison with the median OSCC survival time of Foxp3 staining value

Foxp3 Staining Value	OS		RFS	
	median time (range) (months)	*P* Value	Mean time (range) (months)	*P* Value
1 (*n =* 99)	43 (4–93)	0.000***	39 (2–93)	0.000***
2 (*n* = 106)	38 (2–81)	0.000***	36 (2–59)	0.000***
3 (*n* = 68)	14 (2–56)		11 (1–54)	
	Reference		Reference	

*Abbreviations*: *OS* overall survival, *RFS* relapse free survival

*P* value was determined using Wilcoxon Rank Sum test. *,*P*<0.05;**, *P*<0.01;***, *P*<0.001

## Discussion

In the present study, the expression of Foxp3 was investigated in 273 OSCC tissues by immunohistochemistry. We found that Foxp3 expression was significantly associated with lymph node metastasis and clinical outcome in OSCC. Considering these findings, we suggest that Foxp3 is a potential novel marker for prognosis and represents a therapeutic target for the treatment of OSCC patients.

Continuous effort has been made to identify molecular biomarkers that could provide accurate information regarding OSCC prognosis. While the potential role of tumor Foxp3 as prognostic biomarker of overall survival has been previously investigated, only two studies addressed this issue and draw accordant conclusion that high Foxp3 expression was associated with poor overall survival in patients with TSCC and OHSCC respectively [9, 10]. But the relationship with RFS is not clear. And no association was found with lymph node metastasis.

Takenaka et al. observed that tumor cytoplasm Foxp3 expression was associated with worse relapse-free survival in breast cancer [12]. In small cell lung cancer, relapse- free survival in patients with Foxp3-positive tumor was better with earlier follow-up [13]. Whether Foxp3-positive cancer cells are relevant to recurrence is controversial. For all the head and neck squamous cell carcinoma(HNSCC) types, the relationship of Foxp3 expression with RFS is not clear. This is the first study to describe the association of Foxp3 expression with both five years overall survival (OS) and relapse-free survival (RFS) in OSCC patients. It helps to define the possible link between the biological function of Foxp3 and the progression of OSCC.

Moreover, we provided evidence that Foxp3 expressed by OSCC cells might play a role in the metastasis of OSCC. We found that OSCC patients with high tumor Foxp3 expression had significantly shorter survival time and more lymphnodes involvement than other groups. Although no association was found with lymph node metastasis in the previous TSCC and OHSCC cohort studies, there is still considerable amount of evidence available which indicates Foxp3 expression in breast cancer [14], gastric cancer [6], non-small cell lung cancer [15] and esophageal squamous cell carcinoma [16] is correlated with tumor metastasis. The molecular mechanism of Foxp3 about metastasis remained unclear. One study demonstrated that Foxp3 inhibited breast cancer cell adhesion, invasion and metastasis, while study on the molecular mechanism revealed that Foxp3 inhibited breast cancer metastasis by down-regulating CD44 expression directly [17]. Further studies are required to elucidate its mechanism in OSCC.

## Conclusions

In summary, this study demonstrated that Foxp3 expressed in OSCC cells were significantly correlated with worse prognosis of both overall survival and relapse-free survival in OSCC. Ultimately, these findings provided evidence that Foxp3 expressed by OSCC cells might play a role in the invasion of OSCC, and tumoral Foxp3 might be suitable as a prognostic marker in OSCC. More studies are needed to further explore how tumor cells regulating the metastasis behavior during this interaction, especially in light of the current anticancer efforts to interfere with Foxp3 expression.

## Abbreviations

Foxp3, Forkhead Box P3; HNSCC, head and neck squamous cell carcinoma; IHC, immunohistochemistry; IRS, immunoreactive score; OHSCC, orohypopharynxsquamous cell carcinoma; OS, overall survival; OSCC, oral squamous cell carcinoma; RFS, relapse-free survival; TMAs, tissue microarrays; TSCC, tongue squamous cell carcinoma

## References

[1] Fitzmaurice C, Dicker D (2015). The Global Burden of Cancer 2013. JAMA Oncol.

[2] Siegel R, Ma J, Zou Z, Jemal A (2014). Cancer statistics. CA Cancer J Clin.

[3] Burzyn D, Benoist C, Mathis D (2013). Regulatory T cells in nonlymphoid tissues. Nat Immunol.

[4] Triulzi T, Tagliabue E, Balsari A, Casalini P (2013). FOXP3 expression in tumor cells and implications for cancer progression. J Cell Physiol.

[5] Zuo T, Wang L, Morrison C, Chang X, Zhang H, Li W, Liu Y, Wang Y, Liu X, Chan MW, Liu JQ, Love R, Liu CG, Godfrey V, Shen R, Huang TH, Yang T, Park BK, Wang CY, Zheng P, Liu Y (2007). FOXP3 is an X-linked breast cancer suppressor gene and an important repressor of the HER-2/ErbB2 oncogene. Cell.

[6] Wang L, Liu R, Li W, Chen C, Katoh H, Chen GY, McNally B, Lin L, Zhou P, Zuo T, Cooney KA, Liu Y, Zheng P (2009). Somatic single hits inactivate the X-linked tumor suppressor FOXP3 in the prostate. Cancer Cell.

[7] Ma GF, Miao Q, Liu YM, Gao H, Lian JJ, Wang YN, Zeng XQ, Luo TC, Ma LL, Shen ZB, Sun YH, Chen SY (2014). High Foxp3 expression in tumour cells predicts better survival in gastric cancer and its role in tumour microenvironment. Br J Cancer.

[8] Ladoire S, Arnould L, Mignot G, Coudert B, Rebe C, Chalmin F, Vincent J, Bruchard M, Chauffert B, Martin F, Fumoleau P, Ghiringhelli F (2011). Presence of Foxp3 expression intumor cells predicts better survival in HER2-overexpressing breast cancer patients treatedwith neoadjuvant chemotherapy. Breast Cancer Res Treat.

[9] Liang YJ, Liu HC, Su YX, Zhang TH, Chu M, Liang LZ, Liao GQ (2011). Foxp3 expressed by tongue squamous cell carcinoma cells correlates with clinicopathologic features and overall survival in tongue squamous cell carcinoma patients. Oral Oncol.

[10] Weller P, Bankfalvi A, Gu X, Dominas N, Lehnerdt GF, Zeidler R, Lang S, Brandau S, Dumitru CA (2014). The role of tumour Foxp3 as prognostic marker in different subtypes of head and neck cancer. Eur J Cancer.

[11] Cabrera T, Salinero J, Fernandez MA, Garrido A, Esquivias J, Garrido F (2000). High frequency of altered HLA class I phenotypes in laryngeal carcinomas. Hum Immunol.

[12] Takenaka M, Seki N, Toh U, Hattori S, Kawahara A, Yamaguchi T, Koura K, Takahashi R, Otsuka H, Takahashi H, Iwakuma N, Nakagawa S, Fujii T, Sasada T, Yamaguchi R, Yano H, Shirouzu K, Kage M (2013). FOXP3 expression in tumor cells and tumor-infiltrating lymphocytes is associated with breast cancer prognosis. Mol Clin Oncol.

[13] Tao H, Mimura Y, Aoe K, Kobayashi S, Yamamoto H, Matsuda E, Okabe K, Matsumoto T, Sugi K, Ueoka H (2012). Prognostic potential of FOXP3 expression in non-small cell lung cancer cells combined with tumor-infiltrating regulatory T cells. Lung Cancer.

[14] Merlo A, Casalini P, Carcangiu ML, Malventano C, Triulzi T, Mènard S, Tagliabue E, Balsari A (2009). FOXP3 expression and overall survival in breast cancer. J Clin Oncol.

[15] Dimitrakopoulos FI, Papadaki H, Antonacopoulou AG, Kottorou A, Gotsis AD, Scopa C, Kalofonos HP, Mouzaki A (2011). Association of FOXP3 expression with non-small cell lung cancer. Anticancer Res.

[16] Xue L, Lu HQ, He J, Zhao XW, Zhong L, Zhang ZZ, Xu ZF (2010). Expression of FOXP3 in esophageal squamous cell carcinoma relating to the clinical data. Dis Esophagus.

[17] Zhang C, Xu Y, Hao Q, Wang S, Li H, Li J, Gao Y, Li M, Li W, Xue X1, Wu S, Zhang Y, Zhang W (2015). FOXP3 suppresses breast cancer metastasis through downregulation of CD44. Int J Cancer.

